# The Impact of Hyperphosphatemia on Mineral and Bone Metabolism: Implications for Bone and Vascular Health

**DOI:** 10.3390/ijms27041931

**Published:** 2026-02-17

**Authors:** Nerea González-García, Angie Hospital-Sastre, Sara Fernández-Villabrille, Paula Calvó-García, María Piedad Ruiz-Torres, Carlos Gómez-Alonso, Cristina Alonso-Montes, Manuel Naves-Díaz, Sara Panizo, Natalia Carrillo-López

**Affiliations:** 1 Metabolismo Óseo, Vascular y Enfermedades Inflamatorias Crónicas, Instituto de Investigación Sanitaria del Principado de Asturias (ISPA), 33011 Oviedo, Spain; 2 Redes de Investigación Cooperativa Orientadas a Resultados en Salud (RICORS2040, Kidney Disease), 28040 Madrid, Spain; 3 Unidad de Metabolismo Óseo, Unidad de Gestión Clínica de Medicina Interna, Hospital Universitario Central de Asturias, 33011 Oviedo, Spain; 4 Programa de Doctorado en Ciencias de la Salud, Universidad de Oviedo, 33011 Oviedo, Spain; 5 Physiology Unit, Department of Systems Biology, Facultad de Medicina y Ciencias de la Salud, Universidad de Alcalá, Área 5-Fisiología y Fisiopatología Renal y Vascular del Instituto Ramón y Cajal de Investigación Sanitaria (IRYCIS), 28871 Alcalá de Henares, Spain

**Keywords:** hyperphosphatemia, phosphate homeostasis, CKD-MBD, FGF23, PTH, Klotho, vascular calcification, bone metabolism

## Abstract

Phosphorus is an essential mineral involved in bone mineralization, energy metabolism, and cellular signaling, whose serum concentration is tightly regulated by an endocrine network including fibroblast growth factor 23 (FGF23), parathyroid hormone (PTH), vitamin D and Klotho. Disruption of this balance, particularly in chronic kidney disease (CKD), leads to hyperphosphatemia, which is strongly associated with bone fragility, vascular calcification, and increased mortality. In CKD, impaired phosphorus homeostasis triggers endocrine dysregulation characterized by elevated PTH and FGF23 levels, Klotho deficiency, and altered vitamin D metabolism, resulting in major skeletal and vascular consequences. Experimental and clinical evidence indicates that phosphorus overload contributes directly to skeletal deterioration and early vascular remodeling, even prior to clinically detectable renal impairment. Moreover, high dietary phosphorus intake under conditions of normal renal function reproduces several molecular and structural alterations typically observed in CKD, supporting a pathogenic role for chronic phosphorus excess. The dietary source of phosphorus has gained increasing relevance, as inorganic phosphate additives exhibit high intestinal bioavailability and impose a greater systemic phosphorus burden. Current management strategies rely on dietary restriction, phosphate binders, modulation of intestinal phosphorus transport and optimization of mineral-regulating hormones, although evidence for improved clinical outcomes remains limited. A deeper understanding of the molecular mechanisms linking phosphorus overload to bone and vascular pathology may facilitate the development of more effective preventive and therapeutic strategies.

## 1. Introduction

Phosphorus was first identified in 1669 by Henning Brand during experiments involving human urine [1]. Since this discovery, it has attracted considerable scientific interest, due to its fundamental importance in biological systems, serving as the second most abundant after calcium in the organism, and it comprises approximately 1% of body weight, with an overall content of around 12 g per kilogram [2,3,4]. Physiologically, phosphorus is distributed by the following way: approximately 85% is stored in the mineral matrix of bone and dental tissues, 14% is localized within soft tissues, and the remaining 1% exists in extracellular fluids [5]. Of this 1% of phosphorus, approximately 10% to 20% is bound to proteins, while about 5% forms complexes with cations such as calcium, sodium and magnesium [6]. The remainder predominantly exists in the form of hydrogen phosphate and dihydrogen phosphate, with a typical physiological ratio of 4:1 [6].

Phosphorus is utilised by the body predominantly in the form of phosphate, participating in numerous biochemical processes essential for maintaining homeostasis [7]. It play a critical role in the mineralisation of bone, as it is one of the principal components of hydroxyapatite; as well as in energy metabolism, serving as a key component of adenosine triphosphate (ATP) [3]. Additionally, phosphorus is an integral part of nucleotides, which are crucial for protein synthesis and cellular functions [3]. It contributes to the maintenance of acid-base balance and is necessary for the phosphorylation and activation of various enzymes [3,8]. Moreover, phosphorus is a fundamental component of phospholipids, which are essential for the structure and function of cellular membranes [3].

The principal sources of phosphorus in the diet can be categorised into two forms: organic and inorganic [9]. Organic phosphorus is predominantly found in protein-rich foods derived from animal sources exhibiting superior bioavailability compared to that from plant sources [9]. In contrast, inorganic phosphorus is commonly present in food additives and preservatives and is abundant in processed products such as bottled drinks and frozen foods [10]. The absorption efficiency of inorganic phosphorus in the human body ranges from 80% to 100%, whereas the bioavailability of organic phosphorus is about 60% when derived from animal-based foods, and is 20% to 50% when derived from plant sources, based on the phytate content [9,11,12,13,14]. In recent years, an increase in daily phosphorus intake has been observed, particularly as inorganic phosphorus [15]. This has led to elevated serum phosphorus levels, which may activate homeostatic mechanisms [15]. Such alterations can contribute to the development of bone and cardiovascular pathologies, especially in patients with chronic kidney disease (CKD) [15,16,17,18].

Serum phosphorus concentrations are tightly regulated to preserve homeostasis, typically remaining within a narrow range of 2.5 to 4.5 mg/dL in adults [2]. Levels above 6.5 mg/dL are considered clinically significant hyperphosphatemia with the exception of children in which these levels might be considered as normal [19]. Nevertheless, levels above 4.5 mg/dL are associated with increased all-cause and cardiovascular mortality, particularly among individuals with CKD [20]. This pathological increase in serum phosphorus levels can arise through multiple, often overlapping mechanisms. The principal cause is impaired renal phosphate excretion, typically observed in CKD [21]. Other causes include increased phosphorus intake, phosphate release from cells, hormonal disturbances, vitamin D intoxication and acute or chronic metabolic acidosis [15,22].

To counteract these elevations and maintain mineral balance, the body activates endocrine responses. In this context, parathyroid hormone (PTH), secreted by the parathyroid glands, plays a central role in restoring phosphorus balance by acting on the bones, kidneys, and indirectly on the gastrointestinal system [23]. In bone, PTH stimulates osteoclastic activity, leading to the release of phosphorus into the bloodstream [24,25]. In the kidneys, PTH reduces phosphorus reabsorption in the proximal tubules, thereby promoting its excretion in urine [26]. At the same time, PTH enhances the synthesis of the most active metabolite of the vitamin D hormonal system (calcitriol or 1,25-dihydroxyvitamin D) [27]. Although PTH does not act directly on the gastrointestinal tract, the increase in calcitriol promotes the intestinal absorption of dietary phosphorus [27].

Calcitriol also exerts negative feedback on the parathyroid glands, inhibiting further synthesis and secretion of PTH [28]. In addition, elevated phosphorus levels, along with calcitriol, stimulate the production of fibroblast growth factor 23 (FGF23) in bone [29]. FGF23 suppresses PTH secretion at the parathyroid gland and, in the kidneys, inhibits calcitriol synthesis while promoting phosphate excretion in urine—further contributing to phosphorus homeostasis [30,31].

In individuals with normal renal function—and even in the early stages of CKD—all of these regulatory mechanisms remain physiologically functional [32]. The combined effects of elevated PTH and FGF23 ultimately lead to a reduction in serum phosphate concentrations, thereby maintaining phosphate homeostasis [3].

Homeostasis can become dysregulated with ageing, particularly when renal function is impaired [33]. When renal disease progresses, resulting in enhanced phosphorus reabsorption and a reduction in calcitriol synthesis [34]. Consequently, these changes lead to decreased intestinal calcium absorption, which can have significant implications for overall mineral balance and bone health [35,36]. The elevation in serum phosphate levels combined with reduced levels of calcium and calcitriol stimulates the synthesis and secretion of PTH [28,37,38]. This, in turn, enhances bone resorption, further contributing to the release of phosphorus, but also calcium, into the bloodstream [25,39].

This phosphate overload, in addition to continuously stimulating PTH synthesis and secretion, also induces FGF23 production [40]. Under normal physiological conditions, FGF23 suppresses PTH synthesis [41]. However, in advanced stages of CKD, this regulatory mechanism is impaired due to the downregulation of Klotho, the essential co-receptor for FGF23 in the parathyroid glands [42,43]. As a result, FGF23 is unable to inhibit PTH secretion, contributing to the development of hyperphosphatemia and secondary hyperparathyroidism [31,44]. This dysfunctional state ultimately establishes a maladaptive feedback loop in which persistent elevations of phosphate, rising PTH and increased FGF23—aggravated by Klotho deficiency—perpetuate systemic phosphorus overload and progressive mineral imbalance.

Hyperphosphatemia is a key driver of several pathological processes involved in accelerated ageing, including vascular calcification, impaired bone mineralization and alterations in cellular signalling pathways [45,46,47]. These disturbances are particularly relevant in the context of CKD, where phosphate dysregulation contributes to both cardiovascular complications and skeletal abnormalities [45,48]. Numerous studies have linked elevated serum phosphate levels to increased mortality and cardiovascular events, not only in patients with renal dysfunction but also in general population [7,17,49,50,51,52,53,54]. As these alterations affect both vascular and skeletal systems, understanding the regulation of this process is essential for a better knowledge of mineral and bone disorders associated with kidney disease.

In this review, we will focus on the interplay between phosphorus and other key regulators of bone and mineral metabolism including FGF23, PTH, vitamin D and Klotho on bone and vessel in CKD and also in normal renal physiology. Finally, we will discuss potential strategies for the management of hyperphosphatemia.

## 2. Role of Phosphorus in Chronic Kidney Disease: Interrelation with Other Pathophysiological Factors

### 2.1. Phosphorus and FGF23 in CKD

FGF23 is a member of the fibroblast growth factor (FGF) family and was initially identified through the study of autosomal dominant hypophosphatemic rickets, a condition in which mutations lead to elevated circulating levels of this protein and subsequent hypophosphatemia [55,56]. It is synthesized in bone, primarily by osteocytes and to a lesser extent by osteoblasts, as an inactive precursor that requires processing to generate its mature form [29]. This synthesis is induced by high dietary phosphate levels and vitamin D [29].

FGF23 exerts its biological effects primarily on the kidney, where it decreases the expression of the NaPi2a and NaPi2c cotransporters, thereby increasing renal phosphorus excretion [6,7,29,55,56,57,58,59]. Additionally, it inhibits the renal synthesis of vitamin D by suppressing 1α-hydroxylase and stimulating 24-hydroxylase, which catalyzes the formation of 24,25-dihydroxycholecalciferol, an inactive form of this vitamin [55,59]. FGF23 also acts on the parathyroid gland by inhibiting PTH synthesis; it has a phosphaturic effect and reduces vitamin D levels, which may ultimately lead to hypocalcaemia [6,29,60].

As previously mentioned, Klotho is the specific cofactor of FGF23, conferring this protein its specificity for the kidney and parathyroid gland, which is mediated through the formation of a binary complex between Klotho and the FGF receptor, phenomenon that significantly enhances the receptor’s affinity for FGF23 [6,7,55,56,60,61,62,63,64,65].

Given its central role in phosphorus metabolism and vitamin D regulation, FGF23 has emerged as a critical mediator in the pathophysiological response to hyperphosphatemia. While its systemic effects on renal phosphorus excretion and hormonal balance are well established, increasing evidence suggests that FGF23 also exerts local effects at bone and vascular level. In the context of CKD where hyperphosphatemia is prevalent, these local actions may contribute to bone mineral disorders and vascular calcification. Therefore, understanding the role of FGF23 in both skeletal tissue and vascular system is essential for elucidating the mechanisms underlying CKD-related mineral and bone disorders (CKD-MBD).

#### 2.1.1. Bone System

As previously discussed, hyperphosphatemia stimulates the synthesis and secretion of FGF23 and, together with other factors such as PTH, leads to profound alterations in bone metabolism, particularly affecting bone mineralisation [45]. Consequently, these changes elevate the risk of fractures. Moreover, a positive association was observed between elevated serum levels of FGF23 and increased sclerostin expression, a key inhibitor of the Wnt signaling pathway, which is essential for skeletal development and the maintenance of bone mass [66,67].

In a rat model of CKD, hyperphosphatemia induced by a high-phosphorus diet was accompanied by elevated serum PTH and FGF23, cortical bone loss with increased porosity, and upregulation of both bone formation and resorption markers [68]. These changes were accompanied by increased expression of some Wnt pathway inhibitors, concretely secreted frizzled related proteins (Sfrp) and Dickkopf-1 (Dkk1). As it was previously described in the literature, PTH reduced these inhibitors [69]. However, the combined FGF23 and Klotho treatment again increased Wnt inhibitors, particularly in this study Dkk1, and inactivated Wnt signalling [68]. In addition, in vitro, phosphorus alone did not alter bone markers or Wnt inhibitors [68]. These findings suggest that in CKD, hyperphosphatemia might indirectly upregulate different Wnt inhibitors through FGF23.

A previous study involving juvenile cystic kidney (Jck) mice have reported analogous findings, indicating significant bone remodelling associated with the progression of CKD [70]. These studies observed elevated levels of phosphorus and FGF23, coupled with increased bone turnover. Despite heightened trabecular remodelling, there was a notable reduction in overall bone volume, characterized by increased bone porosity and diminished cortical thickness, a process accompanied by elevated levels of Wnt inhibitors.

Furthermore, numerous clinical studies have observed an elevation in the levels of Wnt inhibitors in bone in renal patients who exhibit increased FGF23 levels, indicating a positive association between these two biomarkers [66,71,72,73,74,75].

#### 2.1.2. Vascular System

Vascular disease is a common complication of CKD patients and the first cause of mortality in this population [76]. An epidemiology study described a strong association between higher FGF23 serum levels and mortality in CKD patients probably due, among others, to vascular complications [77]. Thus, FGF23 has been considered in this last decade as a likely risk factor of the alterations in the vascular system. Within the context of the previously examined rat CKD model, significant vascular calcifications were identified in most of the aortas of these animals [68,78]. These vascular calcifications were characterized by regions of complete mineralization, the presence of osteoid, and the existence of multinucleated cells, suggesting a process similar to bone remodeling at vascular level [78]. Furthermore, an inhibition of genes associated with muscular contractility in vessels was observed, concretely tropomyosin and elastin, alongside an increase in the expression of Wnt pathway inhibitors [78]. This suggests a dedifferentiation of smooth muscle cells in the vasculature toward a bone-like phenotype, which even already exhibits elevated levels of Wnt inhibitors.

It is difficult to prove if FGF23 has an independent effect on the vascular calcification because of the tight relation between phosphorus and FGF23. In pediatric patients undergoing hemodialysis, a significant correlation between P and FGF23 levels and the presence of coronary calcification was observed [79]. Nevertheless, in a detailed analysis of a large CKD cohort of approximately 1500 patients from the Chronic Renal Insufficiency Cohort (CRIC) study, no significant association was found between circulating FGF23 levels and thoracic or coronary arterial calcification scores [80]. Overall, evidence from in vitro studies regarding the role of FGF23 in vascular calcification remains inconsistent.

These discrepancies among studies may be attributed to several critical factors. First, variations in experimental models—ranging from 2D cell cultures to complex ex vivo aortic rings—often yield different results due to the presence or absence of the klotho co-receptor or other systemic mediators [80,81]. Second, the concentrations of FGF23 used in many in vitro investigations are often supra-physiological, which may not accurately reflect the chronic, low-grade exposure seen in early-stage CKD patients [80,81,82]. Furthermore, the role of FGF23 appears to be highly context-dependent, particularly regarding ambient phosphorus levels. Evidence suggests that FGF23 may only exert pro-calcifying or detrimental effects on vascular smooth muscle cells (VSMCs) when synergistic hyperphosphatemia is present, complicating the isolation of its independent vascular impact [80,81].While several reports suggest that FGF23 does not induce vascular calcification, even under high phosphorus conditions; while other investigations report a dose-dependent increase in calcium levels in cultured vascular smooth muscle cells (VSMCs), but this phenomenon is also dependent upon the presence of elevated phosphorus conditions [80,81]. Taken together, these methodological and contextual differences provide a plausible explanation for the conflicting conclusions regarding the vascular effects of FGF23.

In CKD, not only phosphorus and FGF23 are altered, but other factors such as PTH, vitamin D, Klotho, and even uremic toxins can also be dysregulated, making it difficult to separate the independent effects of each factor. To better isolate the role of FGF23, a recent research conducted on rats with normal renal function has demonstrated that the injection of recombinant FGF23 results in a significant decrease in elastin levels [82]. This was accompanied by notable morphological alterations in the VSMCs within the median layer, the disorganisation of the elastic fibers with a significant amount of extracellular matrix surrounding them and an increase in vascular stiffness, as quantified by measuring pulse pressure. To investigate whether the observed alterations might be attributed to FGF23, VSMCs were cultured with high concentrations of FGF23 alongside normal phosphorus levels. The results indicated a transition in the cellular phenotype from a contractile to a “synthetic” phenotype, characteristic of the pre-osteoblastic stage. This transition was evidenced by a reduction in α-actin and elastin expression, alongside an increase in matrix metalloproteinase 9 (MMP9), which is specific to the synthetic phenotype of VSMC.

### 2.2. Phosphorus and PTH in CKD

Parathyroid hormone is secreted by the parathyroid glands [29]. Its synthesis is stimulated by hypocalcemia and hyperphosphatemia, while it is inhibited by vitamin D, FGF23, and elevated serum calcium levels [29]. Concretely, elevated levels of serum phosphorus directly stimulates PTH secretion via inhibition of the calcium-sensing receptor (CaSR) in the parathyroid gland [83]. In the kidney, PTH decreases the expression of the sodium-phosphate cotransporter type IIa (NaPi2a) in the apical membrane of proximal tubular cells by promoting its internalization and downregulating its transcription, thereby enhancing urinary phosphorus excretion [7]. In addition, it stimulates the synthesis of calcitriol through the induction of 1-α-hydroxylase activity [7]. Consequently, PTH increases extracellular calcium concentrations via vitamin D activation, without simultaneously raising serum phosphorus levels, since it promotes phosphorus excretion in parallel [29,60].

#### 2.2.1. Bone System

As previously mentioned, hyperphosphatemia is associated with an increased synthesis and secretion of PTH [83]. This dysregulation can lead to alterations in bone mineralisation, heightening the risk of fractures [84].

A previous study aimed to disentangle the independent contributions of elevated phosphorus and PTH to bone loss and the development of vascular calcification in a CKD rat model [85]. Parathyroidectomy was performed in a subset of animals, followed by implantation of a continuous-release PTH pellet to maintain physiological PTH levels. Consistent with the findings of the previous mentioned study [68], while nephrectomy combined with high dietary phosphorus elevated PTH, phosphorus, and FGF23 levels, parathyroidectomized rats showed normal PTH and a more moderate increase in FGF23 despite comparable hyperphosphatemia. Under these conditions, controlled PTH levels led to marked improvements in cortical bone structure, whereas rats with elevated PTH showed clear alterations in bone remodeling. The concominant reduction in Wnt pathway inhibitors further supports the notion that PTH directly contributes to bone deterioration, independently of serum phosphorus levels.

This phenomenon is well established in the literature, with numerous studies demonstrating the direct effects of PTH on bone metabolism, independent of phosphorus levels. The impact of PTH on bone structure is influenced by its mode of administration; continuous exposure to PTH can induce catabolic effects that promote osteoclastogenesis and enhance bone resorption [86,87,88,89,90,91]. In contrast, intermittent exposure to PTH has been shown to induce anabolic effects, promoting osteoblastic differentiation and stimulating trabecular and cortical bone formation, improving bone microarchitecture and increasing bone strength [65,68,92,93,94,95]. Consequently, intermittent dosing with PTH lowers fracture risk in humans and is authorized in many countries for the treatment of postmenopausal osteoporosis [96,97]. This treatment is also being investigated for its potential to accelerate fracture repair and reduce the incidence of defective bone repair [98].

#### 2.2.2. Vascular System

In the model mentioned before, rats with parallel elevations in phosphorus and PTH showed increased aortic calcium content [85]. In contrast, hyperphosphatemic animals with controlled PTH exhibited a reduction in aortic calcium deposition, accompanied by lower aortic Runx2 and Bmp2 expression and higher α-actin levels, suggesting that PTH also contributes directly—independently of serum phosphorus—to the development of vascular calcification. To further investigate the direct vascular actions of PTH, VSMCs were exposed to varying PTH concentrations under non-calcifying and calcifying conditions. PTH had no effect on calcium accumulation under standard phosphorus levels. However, under calcifying high-phosphorus conditions, high PTH concentrations enhanced calcium deposition, increased Runx2 expression, and suppressed α-actin beyond the effects of phosphorus alone. Conversely, under calcifying conditions, low PTH concentrations (10^−9^ M or lesser) reduced calcium content and Runx2 expression while increasing α-actin. Overall, these findings demonstrate a direct effect of PTH on vascular calcification, but in the presence of high phosphorus concentrations.

Building on these observations, another study with nephrectomized rats receiving continuous physiological replacement of PTH showed that hyperphosphatemia alone, even when associated with elevated Ca×P, did not induce vascular calcification [99]. Further studies confirmed that continuous infusion of high PTH concentrations induced extensive medial aortic and coronary calcification, regardless of calcium, phosphate, or renal status [100]. This underscores that elevated PTH alone is sufficient to drive vascular calcification. In another study where vascular calcification was also observed exclusively in animals receiving supraphysiological PTH infusion [101], phosphate overload and normal PTH infusion were associated with phenotypic changes in VSMCs, including increased Runx2 and type I collagen expression, illustrating the direct osteogenic effect of PTH on vascular cells and supporting its role as a one of the primary drivers of calcification in diverse biochemical contexts.

### 2.3. Phosphorus and Vitamin D in CKD

The active form of vitamin D, 1,25-dihydroxycholecalciferol, is synthesized in the kidney, where the enzyme 1-α-hydroxylase—encoded by the CYP27B1 gene—converts the precursor 25-hydroxycholecalciferol into its mature and biologically active form [102]. The precursor 25-hydroxycholecalciferol is synthesized in the liver from cholecalciferol, which is mainly derived from cutaneous production [29]. Vitamin D activation is stimulated by low serum calcium and phosphorus levels, as well as by PTH, all of which upregulate CYP27B1, whereas high concentrations of phosphorus, calcium and FGF23 inhibit its activity [29].

In the intestine, vitamin D acts on enterocytes to regulate transcellular phosphorus transport through upregulation of the NaPi-IIb cotransporter, thereby increasing dietary phosphorus uptake [6,7,29]. It also enhances calcium absorption across the intestinal epithelium [7]. In bone, vitamin D increases osteoclastic activity, promoting the release of phosphorus and calcium into the circulation [103]. Additionally, it stimulates the synthesis and secretion of FGF23 in osteoblasts and osteocytes [104]. Within the parathyroid gland, vitamin D inhibits both the synthesis and secretion of PTH [29,57].

Collectively, these actions of vitamin D lead to an overall increase in circulating calcium and phosphorus levels.

#### 2.3.1. Bone System

Another consequence of hyperphosphatemia is the reduced synthesis of calcitriol, which—together with calcium deficiency—stimulates PTH secretion [105]. Elevated PTH acts on bone, promoting further phosphorus release, which consequently increases bone fragility and fracture risk, while also contributing to cardiovascular alterations such as the development of vascular calcification, as we previously discussed [106,107].

In a well-known meta-analysis, the daily administration 700–800 IU/day of vitamin D has demonstrated to reduce hip and non-vertebral fracture risk [108]. Consistent with these findings, in patients with early-stage CKD, vitamin D supplementation also supports a beneficial skeletal effect [109]. In this randomized controlled trial, high-dose cholecalciferol effectively corrected vitamin D deficiency, increased circulating vitamin D levels, and significantly reduced intact PTH and bone turnover markers compared with placebo. These data further reinforce the role of vitamin D in improving mineral metabolism and attenuating excessive bone turnover in CKD. However, a recent systematic review including more than 11,000 participants have shown that vitamin D therapy does not clearly reduce mortality, fractures, or cardiovascular events in CKD stages 3–5, despite consistent biochemical improvements [110].

Although active vitamin D is commonly used to control secondary hyperparathyroidism in dialysis patients, its beneficial effect on bone health independently of the suppression of PTH is unknown [111]. A recent study of the Dialysis Outcomes and Practice Patterns Study (DOPPS) suggest a non-beneficial effect of active vitamin D in fracture prevention [112].

#### 2.3.2. Vascular System

In the case of the vascular system, experimental and clinical studies have shown that vitamin D deficiency is associated with an increase in cardiovascular alterations [113,114,115]. However, the use of vitamin D to prevent the development of vascular calcification remains controversial, as elevated levels may also promote calcification. This can be observed in studies where calcitriol increased calcium content in VSMCs in the presence of phosphorus [116], as well as in another work using Von Kossa staining in vivo in animals with CKD, which demonstrated the development of vascular calcification with calcitriol or paricalcitol treatment, although the degree of vascular calcification was lower with paricalcitol [117].

On the other hand, a recent study reported that the treatment with the combination of 25(OH)D_3_ and paricalcitol in mice with CKD led to excessive PTH suppression, hypercalcemia, and hyperphosphatemia [118]. Despite these severe conditions of systemic calcification, the treatment in CKD mice prevented the increase in the osteogenic factor Runx2 and the reduction in aortic miR-145, which is a microRNA responsible for maintaining the contractile phenotype of VSMCs and whose decrease is associated with vascular calcification [119,120]. These findings suggest that vitamin D or some of its analogues may prevent or attenuate the development of vascular calcification by preserving miR-145 levels; however, these results require confirmation, and the controversy remains unresolved.

### 2.4. Phosphorus and Klotho in CKD

Klotho was named after the Greek goddess who wove the thread of destiny [56,62], and it was first identified by Kuro-o and colleagues in 1997 in a mouse strain exhibiting a phenotype resembling human premature aging due to a defect in this gene, though α-Klotho may play a critical role in the regulation of the aging process [55,60,61,65,121]. Klotho is a transmembrane protein with a characteristic structure that includes an N-terminal signal sequence, a single transmembrane helix near the C-terminal region, and an extracellular domain containing two homologous repeats, KL1 and KL2 [65]. It is highly expressed in the distal renal tubules and in the choroid plexus of the brain [56,61,65]. Functionally, Klotho is a multifunctional molecule that can act as a cofactor, enzyme, and ligand [61]. Its extracellular domain can be enzymatically cleaved by the proteases ADAM10 and ADAM17 or generated through alternative splicing, giving rise to a soluble form that circulates in blood, urine, and cerebrospinal fluid, where it exerts additional biological function [56,65]. The membrane-bound α-Klotho acts as an obligate co-receptor for FGF23, playing a central role in phosphorus and vitamin D metabolism, while the soluble form exerts hormone-like effects and regulates ion channels, growth factor receptors, and endocrine FGFs such as FGF19 and FGF21 [122]. Additionally, Klotho regulates renal phosphate handling by modulating the NaPi-IIa and NaPi-IIc transporters in the proximal tubule, thereby contributing directly to phosphaturia [123]. Importantly, it is now recognized that Klotho deficiency represents an early and primary event in the pathophysiology of CKD-MBD rather than a mere secondary consequence of kidney failure [124].

Both aging and CKD are characterized by a progressive decline in soluble Klotho levels, a reduction that appears early in CKD—even before the rise in circulating FGF23 [125,126]. Reduced Klotho levels are already detectable in humans from CKD stages 2–3, reinforcing its role as an early biomarker and causal mediator [125]. This decrease in soluble Klotho may compromise its physiological actions, with subsequent detrimental consequences for bone and vascular health, as it will be discussed below.

#### 2.4.1. Bone System

Regarding bone involvement, studies using mouse models of adenine-induced kidney injury have shown that, as disease progresses, animals develop increasing levels of serum phosphorus, PTH, and FGF23, accompanied by a progressive decline in soluble circulating Klotho [127]. This reduction in soluble Klotho occurs in parallel with trabecular thinning and subsequent loss of bone mass. Whether this bone loss is directly caused by diminished Klotho levels remains difficult to establish. In addition, Klotho deficiency generates resistance to FGF23 both in the kidney and bone, further disrupting mineral metabolism [128]. In the clinical setting of human CKD, the interaction between FGF23 and Klotho within the bone microenvironment is increasingly recognized as a key pathological factor [68,129,130]. Studies in human bone biopsies have demonstrated that as renal function declines, the skeletal expression of Klotho is significantly altered, which correlates with the severity of renal osteodystrophy [62,124,125,129,130,131,132,133]. This suggests that the decline in local Klotho is not merely a consequence but a driver of ‘bone Klotho resistance,’ impairing the physiological signaling of FGF23 in osteocytes and osteoblasts. Consequently, the local disruption of this axis contributes to osteocyte dysfunction and mineralization defects, regardless of systemic mineral levels. Furthermore, evidence in CKD patients shows that the skeletal FGF23–Klotho axis interacts with other local pathways, such as the Wnt/β-catenin signaling, where Klotho deficiency may exacerbate the pro-calcifying and anti-mineralizing effects of elevated FGF23 [68].

Only a limited number of studies have explored the direct effects of soluble Klotho on bone. One such study, conducted in pre-osteoblastic cells, demonstrated that soluble Klotho promotes osteoblastic differentiation—inducing genes such as Runx2, alkaline phosphatase, and osteocalcin—even in the absence of hyperphosphatemia [134]. Furthermore, a study has demonstrated that Klotho expressed within bone is essential for the proper induction of FGF23 during renal injury [129]. Mice with Klotho deletion specifically in long bones fail to upregulate FGF23 in response to uremia, resulting in lower circulating levels of FGF23 and PTH, alongside higher levels of calcium, 1-α-hydroxylase, and 1,25-dihydroxyvitamin D. Whether these effects on osteoblasts and osteoclasts are mediated predominantly through direct local actions of Klotho or through systemic effects remains a matter of debate, and current evidence suggests that both mechanisms contribute to altered bone remodelling [131].

Together, these findings underscore the critical role of Klotho—both circulating and bone-derived—in maintaining bone integrity and mineral homeostasis, highlighting its potential as a therapeutic target to mitigate CKD-related bone and mineral disorders.

#### 2.4.2. Vascular System

Several studies in CKD mouse models have shown that the development of vascular calcification can be prevented by the administration of soluble Klotho. This suggests that Klotho may play a specific role in modulating vascular calcification.

Consistent with this idea, additional evidence demonstrates that Klotho deficiency itself contributes directly to soft-tissue calcification in CKD [135]. In a Klotho-overexpressing mouse model, it was exhibited preserved renal function, enhanced phosphaturia, and markedly reduced vascular calcification compared with wild-type CKD animals. Conversely, Klotho-haploinsufficient CKD mice showed exacerbated renal dysfunction and severe calcification.

Recent studies further indicate that in a CKD mouse model fed with a high-phosphorus diet, characterized by hyperphosphatemia, elevated PTH and FGF23, and reduced soluble Klotho; aortic tissue showed decreased expression of α-actin and sclerostin, reflecting a progressive loss of the contractile phenotype of VSMCs [125]. To directly test the role of soluble Klotho in vascular calcification, VSMCs were exposed to a calcifying medium with high phosphorus in the presence or absence of soluble Klotho. Soluble Klotho effectively prevented the phosphorus-induced osteogenic differentiation of VSMCs, confirming its protective role against vascular calcification under conditions of elevated phosphorus.

Furthermore, there is a study where sustained delivery of circulating klotho reduced hyperphosphatemia and markedly prevented vascular calcification even in Klotho null mice, while also stimulating FGF23 production in an FGFR1 - dependent manner [136]. Mechanistically, Klotho preserves the contractile phenotype of VSMCs and modulates intracellular phosphate handling through effects on channels such as TRPV5 and NaPi transporters, thereby limiting phosphorus-driven VSMC osteogenic conversion [137].

In summary, the accumulated evidence positions Klotho as a central modulator of mineral metabolism whose decline in CKD contributes to disturbances affecting both bone and vascular tissues. Importantly, CKD initiates early molecular alterations, such as Klotho deficiency and VSMC dedifferentiation, which precede overt hyperphosphatemia and calcification, highlighting early pathogenic events [135]. Although important mechanistic gaps remain, current findings suggest that preserving or restoring Klotho function may represent a promising strategy to mitigate the systemic complications associated with CKD progression.

Although calcium is in the center of the complex endocrine network that becomes progressively dysregulated in CKD, particularly in the context of sustained hyperphosphatemia, it has not devoted a specific paragraph due to calcium has been extensively included in the complex interconnexion of phosphorus with the other pathophysiological factors involved.

Collectively, phosphorus overload activates a complex endocrine network involving FGF23, PTH, vitamin D and Klotho, leading to coordinated alterations in bone remodeling and vascular homeostasis. The main effects of phosphorus and its key regulators on bone and vascular systems are summarized in Table 1.

## 3. Phosphorus Metabolism in Normal Renal Function

As previously mentioned, phosphorus is an essential mineral involved in numerous physiological processes, including skeletal mineralization, energy metabolism, and cellular signaling [142]. While disturbances in phosphorus homeostasis have been extensively studied in the context of CKD, where hyperphosphatemia is strongly linked to cardiovascular complications and bone disorders, the effects of high dietary phosphorus intake under conditions of normal renal function are less well understood. Understanding these effects is critical for elucidating the systemic consequences of chronic phosphorus excess in otherwise healthy individuals and for identifying potential early interventions to prevent skeletal and vascular complications. An emerging topic in this field is the relevance of the dietary source of phosphorus: organic phosphorus has a lower absorption rate, whereas inorganic phosphorus from additives has a bioavailability exceeding 90%, posing a higher systemic burden even in individuals with normal renal clearance, a particular further problem in industrialized societies where there are an abused intake of aliments with additives [143].

### 3.1. Bone System

An increasing number of studies are examining the effects of high-phosphorus intake in models with normal renal function in bone. It is well stablished that a high dietary phosphorus load reduces bone mass, primarily through secondary elevations in PTH [138]. In one study, rats were fed diets containing 0.6%, 1.2%, or 1.8% phosphorus. Animals receiving the higher-phosphorus diets (1.2% and 1.8%)—which showed the greatest increases in serum PTH—exhibited reduced trabecular area and depth, decreased osteoblastic perimeter, and an increased number of osteoclasts [139]. However, it remains unclear whether these changes are a direct consequence of phosphorus exposure or are indirectly mediated through increased PTH levels.

Clinical studies have demonstrated that a high dietary phosphorus intake is associated with an increased risk of fractures. In the Brazilian Osteoporosis Study (BRAZOS), a 100 mg increase in daily phosphorus consumption was associated with a 9% rise in fracture risk among individuals over 40 years of age [140]. Similarly, in adolescents, the intake of carbonated beverages—characterized by a high phosphorus content—was linked to a threefold increase in fracture risk [144]. In an experimental study designed to assess the effects of a high-phosphorus diet on bone mass and the RANKL/OPG/LGR4 pathway in rats with normal renal function, excessive phosphorus intake resulted in reduced bone mass and appeared to modulate this signaling pathway independently of PTH [119].

Additionally, long-term studies have evaluated the systemic impact of high phosphorus intake in healthy adult animals. In mice fed a high-phosphorus diet (1.2%) for one year, plasma phosphorus, PTH, and calcitriol levels increased, whereas FGF23 remained unchanged [145]. Despite the absence of major renal dysfunction, animals displayed increased bone resorption, lower total and cortical bone mineral density, and a more acidotic internal environment. Importantly, these skeletal alterations occurred independently of the concomitant rise in PTH, suggesting that chronic phosphorus excess exerts direct deleterious effects on bone. Overall, high phosphorus intake in the absence of kidney disease reproduces several of the early molecular and structural bone alterations typically associated with CKD, demonstrating that phosphorus overload per se may initiate pathogenic pathways before renal dysfunction becomes evident [119].

### 3.2. Vascular System

To further investigate the role of high phosphorus intake in vascular calcification under conditions of normal renal function, in a rat model with normal renal function, an 18-week high-phosphorus diet induced significant increases in serum phosphorus and PTH despite preserved renal clearance [141]. This was accompanied by reduced aortic and circulating miR-145 and decreased α-actin expression, without increases in aortic calcium or osteogenic markers, indicating an early stage of vascular calcification. In vitro, elevated phosphorus—but not PTH—reduced miR-145 and α-actin and increased calcium deposition, while PTH enhanced calcification only at 3 mM P and did not induce dedifferentiation. Overall, high phosphorus intake promotes early VSMC dedifferentiation independently of PTH, preceding the emergence of osteogenic features.

However, as it was mentioned before, there are studies that showed that, under normal phosphorus conditions, recombinant FGF23 decreases elastin levels, disrupts elastic fiber organization, and increases vascular stiffness in rats, and that high FGF23 induces a contractile-to-synthetic transition in cultured VSMCs, characterized by reduced α-actin and elastin and increased MMP9 expression [82].

Together, these findings indicate that both elevated phosphorus and FGF23 contribute to early VSMC phenotypic changes and vascular remodeling, establishing a pro-calcifying environment that precedes overt osteogenic transformation.

**Figure 1:**
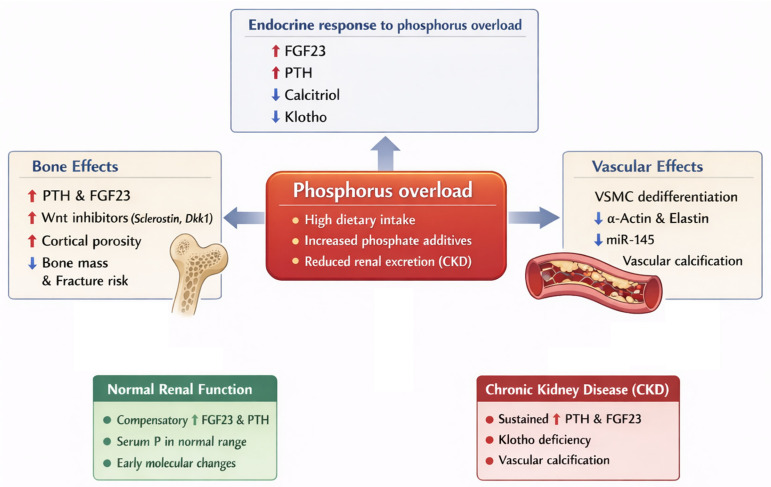
Phosphorus overload as a central driver of bone and vascular alterations. Excess phosphorus intake or reduced renal excretion activates a complex endocrine network involving FGF23, PTH, vitamin D and Klotho. Under conditions of normal renal function, compensatory mechanisms maintain serum phosphate levels within the physiological range, although early molecular alterations in bone and vascular tissues may already occur. In chronic kidney disease, progressive Klotho deficiency and endocrine resistance lead to sustained hyperphosphatemia, impaired bone remodeling and vascular smooth muscle cell phenotypic transition, ultimately promoting bone fragility and vascular calcification.

To provide an integrated view of the systemic impact of phosphorus overload under conditions of normal renal function and chronic kidney disease, Figure 1 depicts a conceptual model linking endocrine regulation with bone and vascular alterations.

## 4. Strategies for the Management of Hyperphosphatemia

The principal recommendation for the management of hyperphosphatemia is dietary phosphorus restriction, which involves reducing the intake of phosphorus-rich foods [146,147]. Patients should avoid excessive consumption of foods high in phosphorus such as dairy products, processed meats, soft drinks, and nuts [146,147]. Processed foods should also be limited, as they often contain phosphorus-based additives that are highly absorbable [148]. Careful label reading is essential to identify these additives in commercial products [149]. Taking all of this into account, it is also important to balance protein requirements to prevent malnutrition [146,147]. It is also relevant to consider the chemical form of dietary phosphorus, as inorganic additives have much higher intestinal absorption than organic animal or plant-based sources [148]. This highlights the value of replacing processed foods with fresh alternatives rather than simply reducing total intake.

In patients with CKD or other conditions associated with hyperphosphatemia, dietary phosphorus restriction remains a key therapeutic measure [150]. A typical Western diet provides around 1500 mg of phosphorus per day and about 1200 mg it is absorbed in the gut; while for patients with kidney failure, the usual recommendation is to limit daily phosphorus intake to about 900 mg per day [151,152]. In a study of 134 hyperphosphatemic patients in End-Stage Kidney Disease, those who received individualized guidance to replace processed foods containing phosphorus additives with similar additive-free options showed a significant reduction in serum phosphorus after 90 days [153]. Nearly 70% of these patients achieved target phosphorus levels, compared with only 18.5% in the control group. Importantly, nutritional status and protein/energy intake remained unchanged in both groups. The findings indicate that substituting foods with phosphorus additives for additive-free alternatives effectively lowers serum phosphorus without compromising nutrition in the patients.

However, as CKD progresses, dietary control alone may not be sufficient, and patients may need pharmacological intervention, which primarily involves oral phosphate binders [154]. Phosphate binders can be classified in calcium-based, non-calcium-based and aluminium-based binders [155]. The use of phosphate binders with or without calcium binders should be a clinical decision taking into account serum calcium levels in the patients. The historical concern regarding aluminium-containing binders has largely been resolved, however, it is important to take into consideration in places with high levels of aluminium in the water for human consumption [156]. Nevertheless, there exist novel and adjunctive therapies that include agents targeting intestinal phosphate absorption [155,157].

Beyond dietary restriction, current therapeutic approaches are evolving towards strategies aimed not only at lowering serum phosphate, but also at modulating intestinal absorption and cellular phosphate handling. Calcium-based phosphate binders remain effective but may promote hypercalcemia and vascular calcification, therefore non-calcium-based alternatives (e.g., sevelamer, lanthanum, bixalomer) are increasingly preferred, particularly in patients with cardiovascular risk or elevated Ca×P product [158,159]. Emerging treatments extend beyond traditional binders: Tenapanor, an inhibitor of the intestinal Na^+^/H^+^ exchanger, has demonstrated significant reductions in phosphate absorption in CKD-5D patients [21,160]. Additional agents—including NaPi-IIb transport inhibitors, nicotinamide and niacin—are under investigation as complementary therapies capable of reducing intestinal phosphate uptake [147,157]. Evidence is also growing regarding the influence of gut microbiome composition on phytate degradation and phosphate bioavailability, providing another potential modifiable target [21]. Overall, these findings support a multifaceted management strategy integrating dietary control, binder therapy, modulation of intestinal transport, mineral balance regulation (PTH–FGF23–Klotho) and possibly microbiota-directed interventions. Further long-term data are required to determine whether these combined therapies improve mortality, vascular calcification progression and enhance clinical outcomes [147,158,159].

In more advanced stages, dialysis may also be necessary to maintain appropriate serum phosphorus levels [21]. Phosphorus is removed during dialysis, and both the dialysis modality and the treatment prescription influence the extent of its clearance. Although predicting phosphorus removal in peritoneal dialysis is challenging, in hemodialysis it can be improved by extending the duration of the sessions and increasing their frequency [155,161].

It is important to note that phosphorus-management strategies should be individualized, considering clinical context, cost-effectiveness, and patient tolerability [146,147,154,158]. Despite the availability of multiple therapeutic approaches, current evidence is insufficient to demonstrate improvements in hard clinical outcomes, highlighting the need for further research in this area [21,146,147,158].

## 5. Final Remarks

Phosphorus is a key regulator of bone and mineral metabolism, and even subtle changes in its balance can activate a complex network of responses involving FGF23, PTH, vitamin D and Klotho. Under conditions of normal renal function, these compensatory mechanisms are pathways maintain serum phosphorus within a narrow range. However, as kidney function declines, these regulatory pathways progressively lose efficiency. This loss of control contributes to hallmark complications of CKD-MBD, including bone abnormalities and vascular calcification.

It is important to acknowledge that much of the current mechanistic understanding of phosphorus-induced pathology relies heavily on experimental animal models, particularly rodents. While these models have been instrumental in identifying key pathways such as the FGF23–Klotho axis, they present significant translational challenges. Species-specific differences in phosphorus handling, bone remodeling rates, and vascular anatomy can limit the direct applicability of these findings to human pathology. Furthermore, experimental conditions often involve acute or supra-physiological mineral loads that may not fully replicate the chronic, multi-factorial progression of CKD-MBD in humans. Therefore, while animal studies provide a vital foundation, more robust longitudinal clinical studies and human tissue analysis are essential to validate these molecular mechanisms and ensure their safe translation into clinical therapies.

Despite substantial advances in our understanding of phosphorus homeostasis, mechanistic and integrative studies are still insufficient to fully explain how these processes interact and become disrupted in CKD. A clearer and more comprehensive understanding of these interactions is essential for accurately interpreting the pathophysiology of CKD-MBD. Such knowledge may also guide the refinement of therapeutic strategies to manage hyperphosphatemia and prevent its long-term complications.

In this clinical context, a major challenge remains in balancing the use of vitamin D and its analogs to suppress PTH levels while avoiding the concomitant risk of hypercalcemia and hyperphosphatemia, which can promote vascular calcification. Therefore, a more integrated understanding of these pathways is essential to define therapeutic windows that protect skeletal integrity without compromising cardiovascular health.

Beyond current pharmacological approach, emerging therapeutic frontiers offer promising strategies for better management of hyperphosphatemia-related disorders. The development of Klotho agonists or the administration of soluble Klotho represents a potential breakthrough to restore the FGF23–Klotho physiological axis and mitigate both vascular calcification and bone loss. Additionally, microRNA (miRNA) targeting has surfaced as a novel epigenetic tool; specific miRNAs involved in osteoblastic transdifferentiation of vascular smooth muscle cells could be modulated to prevent arterial media calcification. These innovative therapies, along with the development of more potent and specific phosphate binder and transporters inhibitors, could redefine the treatment landscape of CKD-MBD, moving towards more targeted interventions that go beyond merely lowering serum phosphorus levels.

Strengthening this knowledge could ultimately support more effective and individualized treatments and improve clinical outcomes for patients living with chronic kidney disease.

## Figures and Tables

**Table 1:** Effects of phosphorus overload and its main regulators on bone and vascular systems.

Factor	Mechanisms Associated with Phosphorus Overload	Bone Effects	Vascular Effects	Relevance in CKD
Phosphorus (direct)	High dietary intake; reduced renal excretion [15]	Reduced bone mass, impaired mineralization, increased fracture risk [138,139,140]	Induction of VSMC osteogenic differentiation [141]	Early driver of CKD-MBD; inorganic phosphate particularly harmful [46]
FGF23	Increased dietary phosphorus; vitamin D stimulation [29]	Increased Wnt pathway inhibitors (sclerostin, Dkk1); altered bone remodeling [68]	Promotes VSMC dedifferentiation and vascular stiffness (context-dependent) [82]	Markedly elevated circulating levels; FGF23 resistance due to Klotho deficiency [127,128]
Parathyroid hormone (PTH)	Hyperphosphatemia; hypocalcemia; reduced vitamin D [29]	Continuous exposure leads to bone resorption and cortical bone loss [86,87,88,89,90,91]	Promotes vascular calcification in the presence of high phosphorus [85]	Secondary hyperparathyroidism is a major contributor to CKD-MBD [31,44]
Vitamin D (Calcitriol)	Suppressed by FGF23 and phosphorus overload [57]	Modulates mineral balance and bone turnover; fracture protection remains controversial [108,109,110]	Dual effects: protective or pro-calcifying depending on dose and context [116,117,141]	Widely used in CKD, but benefits on hard outcomes remain unclear [109,110]
Klotho	Downregulated early in CKD [124]	Supports osteoblast differentiation and bone quality [134]	Maintains VSMC contractile phenotype; inhibits phosphate-induced calcification [125]	Early pathogenic factor and biomarker of CKD progression [135]

Abbreviations: CKD, chronic kidney disease; CKD-MBD, chronic kidney disease–mineral and bone disorder; FGF23, fibroblast growth factor 23; PTH, parathyroid hormone; VSMC, vascular smooth muscle cell.

## Data Availability

No new data were created or analyzed in this study.
